# Grassland invaders and their mycorrhizal symbionts: a study across climate and invasion gradients

**DOI:** 10.1002/ece3.917

**Published:** 2014-02-19

**Authors:** Rebecca A Bunn, Ylva Lekberg, Christopher Gallagher, Søren Rosendahl, Philip W Ramsey

**Affiliations:** 1 Department of Environmental Sciences, Huxley College, Western Washington UniversityES Bldg. 522, 516 High St., Bellingham, Washington, 98225-9181, USA; 2 MPG Ranch1001 S Higgins Ave, Suite A3, Missoula, Montana, 59801, USA; 3 Department of Ecosystem and Conservation Sciences, University of MontanaMissoula, Montana, 59802, USA; 4 Department of Biology, University of CopenhagenOle Maaløes Vej 5, DK-2200, Copenhagen, Denmark

**Keywords:** 454-sequencing, arbuscular mycorrhizal fungi, *Centaurea stoebe*, community structure, plant invasion, plant–soil interactions, *Potentilla recta*

## Abstract

Controlled experiments show that arbuscular mycorrhizal fungi (AMF) can increase competitiveness of exotic plants, potentially increasing invasion success. We surveyed AMF abundance and community composition in *Centaurea stoebe* and *Potentilla recta* invasions in the western USA to assess whether patterns were consistent with mycorrhizal-mediated invasions. We asked whether (1) AMF abundance and community composition differ between native and exotic forbs, (2) associations between native plants and AMF shift with invading exotic plants, and (3) AMF abundance and/or community composition differ in areas where exotic plants are highly invasive and in areas where they are not. We collected soil and roots from invaded and native forb communities along invasion gradients and in regions with different invasion densities. We used AMF root colonization as a measure of AMF abundance and characterized AMF communities in roots using 454-sequencing of the LSU-rDNA region. All plants were highly colonized (>60%), but exotic forbs tended to be more colonized than natives (*P* < 0.001). We identified 30 AMF operational taxonomic units (OTUs) across sites, and community composition was best predicted by abiotic factors (soil texture, pH). Two OTUs in the genera *Glomus* and *Rhizophagus* dominated in most communities, and their dominance increased with invasion density (*r* = 0.57, *P* = 0.010), while overall OTU richness decreased with invasion density (*r* = −0.61, *P* = 0.006). Samples along *P. recta* invasion gradients revealed small and reciprocal shifts in AMF communities with >45% fungal OTUs shared between neighboring native and *P. recta* plants. Overall, we observed significant, but modest, differences in AMF colonization and communities between co-occurring exotic and native forbs and among exotic forbs across regions that differ in invasion pressure. While experimental manipulations are required to assess functional consequences, the observed patterns are not consistent with those expected from strong mycorrhizal-mediated invasions.

## Introduction

Non-native plant invasions can reduce plant diversity and alter ecosystem processes (Levine et al. 2003). Despite substantial research efforts (Richardson and Pyšek 2008), we still lack a predictive understanding of how some exotic plants establish and outcompete native plants. The two most popular hypotheses state that exotic plants become invasive due to an escape from natural enemies in their native range (enemy release hypothesis; Keane and Crawley 2002) or fail to become invasive because they encounter new enemies in their exotic range (biotic resistance hypothesis; Elton 1958).

Mutualists may be equally important for driving plant invasions. For example, invasions may fail due to an absence of specific mutualists in the exotic range (Richardson et al. 2000) or succeed because the invader either brings its mutualists or is able to associate with native or cosmopolitan mutualists (Dickie et al. 2010). It has been suggested that some invasions are successful due to encounters with new, and better, mutualists (Reinhart and Callaway 2006) although solid experimental data for this are lacking. Invasive plants may also disrupt native mutualisms and decrease the competitiveness of the community being invaded (Stinson et al. 2006). Overall, a better understanding of how plant invasions affect – and are affected by – mutualists is critical for improving our understanding of how some exotic plants become invasive.

Here, we focus on arbuscular mycorrhizal fungi (AMF) in the phylum Glomeromycota that form a symbiosis, arbuscular mycorrhiza (AM), with the majority of land plants in which the fungi receive photosynthate in exchange for phosphorus, nitrogen, and other putative services (Smith and Read 2010). Due to their ubiquity and location in the root–soil interface, AMF have been referred to as keystone mutualists (O'Neill et al. 1991) with the potential to influence ecosystem processes such as productivity, carbon and nutrient cycling, water use, and soil structure (Rillig 2004). In general, perennial, coarse-rooted plants appear most dependent on AM (Wilson and Hartnett 1998), but responses are contingent on the particular plant–fungus combination (Klironomos 2003) and environmental conditions (Johnson et al. 1997; Johnson and Graham 2012). Evaluating the likelihood that AMF mediate plant invasions includes assessments of potential differences in the mycorrhizal dependency of the invading exotic and native plants, as well as of invasion-driven changes in AMF abundance and community composition (Pringle et al. 2009).

Previous work has shown that AMF have the potential to influence plant invasive success. For example, the successful invaders *Euphorbia esula* (spurge) and *Centaurea jacea* (knapweed) have a high AM dependency (Klironomos 2002) and *Centaurea stoebe* becomes more competitive toward native plants when grown with AMF (Marler et al. 1999). Both invaders also increase the overall abundance of AMF and harbor different AMF communities than adjacent native grasslands (Lekberg et al. 2013). Another mycotrophic invader, *Solidago canadensis*, alters AMF communities in ways that promote its own growth more than a competing native plant (Zhang et al. 2010). In contrast, *Allaria petiolata* (garlic mustard) and *Bromus tectorum* (cheatgrass) are nonmycorrhizal or have a low mycorrhizal dependency (Stinson et al. 2006; Busby et al. 2011). These two invaders reduce the overall abundance of AMF and the root colonization of mycotrophic native plants (Stinson et al. 2006; Lekberg et al. 2013), which could promote further invasions by reducing native plant competitiveness (Vogelsang and Bever 2009).

Because most experiments involving AMF and plant invasions have been conducted under controlled greenhouse conditions (e.g., Marler et al. 1999; Zhang et al. 2010), it is unclear whether responses are realized in the field in the presence of multiple interacting factors (Smith and Read 2010; Johnson and Graham 2012; but see Callaway et al. 2004). Observational studies incorporate all the complexity of the real world and are capable of revealing the end result of long-term ecological processes in ways that short-term greenhouse studies cannot (Diamond 1983). For example, survey data revealed a likely co-invasion by *Pinus contorta* and its ectomycorrhizal fungi in New Zealand as well as *P. contorta's* greater reliance on cosmopolitan mutualists relative to native plants (Dickie et al. 2010). Taking a similar approach, we assessed the potential for AM to influence the invasion success of *C. stoebe* and *Potentilla recta* by asking whether (1) the exotic forbs harbor a different AMF abundance and/or community composition than native forbs, (2) mycorrhizal associations in natives are altered in the presence of *P. recta* (i.e., assessing the ability of *P. recta* to interrupt native mycorrhizas), and (3) AMF abundance and/or community composition differs between areas where the two forbs are highly invasive and areas where they are not. The observed patterns of AM were examined for consistency with patterns that might be expected in a mycorrhizal-mediated invasion. For example, increases in colonization or changes in composition of the AMF community in more densely invaded plots (presumably toward a stronger association with, or a more beneficial suite of symbionts as in Zhang et al. 2010) would be consistent with mycorrhizal-mediated invasion. We want to stress, however, that unaltered AMF abundance and community compositions does not *disprove* mycorrhizal-mediated invasion, as AM function can vary with abiotic parameters and host plant identity. Further, we did not manipulate our factor of interest (level of invasion by *P. recta* and *C. stoebe*) and controlled experiments are therefore required to assess causation as well as any functional consequences of differences observed (Gotelli and Ellison 2013).

## Materials and Methods

### Site selection and plant community surveys

We used three different sets of survey data to address our questions. First, we examined AMF in *C. stoebe* and *P. recta* and nearby native forbs to determine whether they harbored different abundance and/or AMF communities. We restricted our study to only include mycorrhizal forbs to avoid confounding our comparison of exotics versus natives with plant functional group or mycorrhizal status, which are the most important predictors of mycorrhizal effects (Hoeksema et al. 2010). Next, we sampled across *P. recta* invasion gradients to assess the ability of the invasive *P. recta* to modify the native AM. Third, we compared the AMF abundance and community composition associated with *C. stoebe* and *P. recta* on one ranch in southwestern Montana where the two species are highly invasive, to those in western Washington, where the same two species are only moderately invasive (Gallagher 2011).

To address questions 1 and 2, we identified three sites on MPG Ranch located south of Missoula, Montana (MPG1, 46.677^o^N, 113.987^o^W; MPG2, 46.678^o^N, 113.989^o^W; and MPG3, 46.690^o^N, 113.984^o^W). Each site harbored *P. recta* and *C. stoebe* monocultures, mixed native grass and forb communities, and transition areas in which native and *P. recta* plants were growing side by side. Distance among sites ranged from 250 m to 1500 m, and within each site, all plant community types were within a 50 × 50 m area, which reduced spatial bias.

To address question 3, we located four additional sites near Bellingham in western Washington in which the exotic forbs were found in low abundance: three *C. stoebe* sites (WW1, 48.908^o^N, 122.755^o^W; WW2, 48.787^o^N, 122.708^o^N; and WW3, 48.848^o^N, 122.416^o^W) and one *P. recta* site (WW4, 48.841^o^N, 122.305^o^W). We also included one *C. stoebe* site located in Cheney, eastern Washington (EW1, 47.742^o^N, 117.753^o^W), approximately midway between MPG Ranch and Bellingham. These sites occur along a gradient of climate and invasion density with the highest rainfall, mildest temperatures, and lowest severity of invasion in western Washington.

All 17 plant communities (seven *C. stoebe* invasions, four *P. recta* invasions, three native forb communities, and three transition communities harboring co-occurring native forbs and *P. recta*) were surveyed in June of 2011 by establishing five, 4 m by 4 m plots in each community for a total of 85 plots. Plots within sites were separated by at least 3 m. Within each plot, percent cover of focal forb species was measured in sixteen 1 m by 1 m adjacent sections and averaged to determine mean percent cover (Table 1). Because plant invasions are inherently patchy, invaded areas were separated by native and transition zones at MPG sites (where we sampled from multiple plant community types per site), which reduced the risk of spatial autocorrelation.

**Table 1:** AM colonization and community metrics arranged by survey question and site-plant community plot designation including colonization means and standard deviations (*n* = 5), OTU diversity (Shannon-Weaver), richness (OTU number present), and dominance (percentage of OTU sequences identified as OTU 1 or 16). Cover by exotic is average percent of area covered by the exotic forb in each community; remaining area was covered with grasses, native forbs, and/or bare ground (*n* = 5). Statistical comparisons are summarized in text.

	Site	Plant community	Cover by exotic (%)	AMF colonization^1^			Community metrics^2^		
				Vesicles	Arbuscules	Total	Diversity	Richness	Dominance (%)
Montana: Native versus Exotic Invasions									
	MPG1	*Centaurea stoebe*	52	0.15 ± 0.09	0.24 ± 0.16	0.87 ± 0.08	1.74	10	52
		*Potentilla recta*	82	0.08 ± 0.02	0.15 ± 0.05	0.83 ± 0.08	1.71	7	58
		Native	1	0.12 ± 0.12	0.47 ± 0.19	0.89 ± 0.10	2.00	11	12
	MPG2	*C. stoebe*	43	0.11 ± 0.05	0.26 ± 0.12	0.95 ± 0.05	1.39	8	76
		*P. recta*	55	0.13 ± 0.05	0.03 ± 0.11	0.96 ± 0.03	1.74	9	58
		Native	0	0.06 ± 0.08	0.29 ± 0.08	0.61 ± 0.09	2.11	14	55
	MPG3	*C. stoebe*	51	0.07 ± 0.05	0.29 ± 0.08	0.85 ± 0.08	nd	nd	nd
		*P. recta*	73	0.09 ± 0.07	0.17 ± 0.09	0.85 ± 0.08	1.56	8	68
		Native	0	0.11 ± 0.07	0.23 ± 0.15	0.62 ± 0.22	1.86	10	58
Montana: Transition Zones									
	MPG1	*P. recta*	14	0.06 ± 0.02	0.30 ± 0.15	0.86 ± 0.06	1.79	12	63
		Native	14	0.08 ± 0.07	0.30 ± 0.13	0.70 ± 0.20	2.05	10	45
	MPG2	*P. recta*	20	0.07 ± 0.02	0.26 ± 0.07	0.94 ± 0.03	1.61	9	72
		Native	20	0.11 ± 0.08	0.38 ± 0.08	0.85 ± 0.09	1.98	13	56
	MPG3	*P. recta*	21	0.09 ± 0.08	0.15 ± 0.08	0.76 ± 0.11	1.61	11	67
		Native	21	0.15 ± 0.14	0.21 ± 0.08	0.74 ± 0.12	1.44	10	78
Washington: Exotic Forbs									
Eastern	EW1	*C. stoebe*	17	0.16 ± 0.10	0.31 ± 0.07	0.91 ± 0.05	1.66	8	40
Western	WW1	*C. stoebe*	4	0.04 ± 0.02	0.20 ± 0.08	0.68 ± 0.09	1.72	9	25
	WW2	*C. stoebe*	9	0.10 ± 0.07	0.18 ± 0.10	0.85 ± 0.12	2.08	11	29
	WW3	*C. stoebe*	4	0.14 ± 0.08	0.18 ± 0.08	0.85 ± 0.09	2.08	11	40
	WW4	*P. recta*	25	0.08 ± 0.02	0.14 ± 0.03	0.68 ± 0.15	1.25	6	60

1 Statistical comparisons of AMF colonization are presented in the text.

2 Because insufficient sequences were recovered from *C. stoebe* at MPG3, sample sizes were too small for statistical comparisons.

### Sampling

Four plants were randomly selected and pooled from each of the five plots within each community, resulting in a total of 100 samples (35 *C. stoebe* invasion samples, 20 *P. recta* invasions samples, 15 *P. recta* transition samples, 15 native forb samples, 15 native transition samples). Native forbs included representative native species identified in the survey (Table S1 in Supporting Information), whereas samples from the *P. recta* and *C. stoebe* communities only included the focal species. *Potentilla gracilis*, a native *Potentilla*, was found at MPG3, and five plants were pooled into one sample to qualitatively assess whether the AMF community was more similar to the invasive *P. recta* than to the native forbs.

Plants were carefully removed from the soil with the roots intact, placed in plastic bags, and stored at 4°C for no more than 3 days prior to processing. Fine roots were sampled randomly from each of the four samples per plot for visual quantification of fungal colonization and molecular analyses (approximately 3 mL). Half of the roots were lyophilized and stored at −20°C prior to DNA extraction. We used AM colonization as an indirect measure of AMF abundance and AMF dependency, because intraradical root colonization has been shown to reflect differences in extraradical fungal abundance (and thus total abundance, Lekberg et al. 2013) and extent of root colonization is positively correlated with mycorrhizal responsiveness (Wilson and Hartnett 1998) albeit with a rather low r-value (0.41). Roots for AM colonization analysis were cleared with 2.5% KOH for 48 h, acidified with 3% HCl for 12 h, stained with 0.05% Trypan Blue for 12 h, and destained in distilled water at 4°C (modified method of Phillips and Hayman 1970). Colonization was determined via the magnified intersections method (McGonigle et al. 1990) by examining at least 120 intersections of twenty-four, randomly selected, 1-cm root segments at 200× for arbuscules, vesicles, and hyphae (Nikon Eclipse 80i, Melville, NY). Roots for molecular analysis were lyophilized and stored at −20°C prior to DNA extraction.

Soil samples were collected from the rooting zone of each plant (0–15 cm depth), sieved through a 2-mm sieve, and ultimately pooled within each plant community for a total of 17 soil samples (one from each plant community). Soil was air-dried prior to analysis for texture (Environmental Biogeochemistry Laboratory, University of Montana, Missoula, MT), pH, conductivity, macronutrients and micronutrients (Texas AgriLife Extension Service, Soil, Forage and Water Testing Laboratory, College Station, TX), and soil organic matter based on loss of ignition (Soil Biogeochemistry Laboratory, University of Montana Missoula; Table S2).

### DNA extraction and PCR

We extracted and amplified DNA from each of the five plots per community type separately, and *P. recta* samples were separated from native forbs in transition zones, for a total of 100 samples. DNA extractions were carried out on freeze-dried, ground roots (15 mg) using MoBio PowerPlant DNA isolation kits following the manufacturer's instructions with the double clean-up step (Mo Bio Laboratories, Inc., Carlsbad, CA). Optimized template concentrations (1:10–1:100) were amplified with the LSU rRNA primer set FLR3 and FLR4 (Gollotte et al. 2004), which targets the nLSU-D2 region that is suitable for high-throughput sequencing (Stockinger et al. 2010). We added linkers A and B, and MID-tag sequences (one for each plant community) to primers according to Roche recommendations. The following thermocycling parameters were used: 1 min at 95°C, 34 cycles of 1 min at 95°C, 1 min at 57°C, and 1 min at 72°C with a final extension phase of 5 min at 72°C. The five samples within each plant community were pooled based on band intensity run on a 2% agarose gel. This within-plant community pooling was justified because our replicate was the plant community, not individual plots within each plant community. We purified the 425-to 475-bp bands of the pooled PCR products using the QIAEX II kit (Qiagen, Valencia CA) and eluted with 20 *μ*L 10 mmol/L Tris buffer. Samples from all plant communities (all with different MID-tags) were combined prior to sequencing based on DNA concentrations (nanodrop) to ensure similar representations from all sites prior to sequencing at the Genome Sequencing and Analysis Facility at University of Texas Austin.

### Sequence analyses and OTU designation

We removed sequences less than 200 bp and with a quality score <25. To standardize the number of reads, we resampled 169 sequences from each site (the lowest number of sequences from one site). One *C. stoebe* community (MPG3) was excluded due to very low sequence numbers. OTU accumulation curves, generated using the “specaccum” function with the “rarefaction” method in the vegan package of R (Oksanen et al. 2011), indicated that sequence numbers were sufficient to identify dominant taxa in our samples within the restrictions of our PCR protocol (Fig. S1). Raw sequence files for all sites are accessible at the National Center for Biotechnology Information (NCBI) Sequence Read Archive. We divided the remaining sequences into preliminary OTUs using the cd-hit-est clustering algorithm at 97% similarity (default settings, except aS = 0.8) and blasted representatives from each cluster against NCBI GenBank. All non-AMF clusters and AMF singletons were removed to reduce the risk of artificially inflating richness due to sequencing errors (Dickie 1983). We used a neighbor joining tree to identify OTUs as monophyletic clades, blasted representative sequences against NCBI GenBank to obtain the most similar sequences from other studies (Table S3), and counted their relative read numbers (Table S4). A neighbor Net was constructed on all remaining sequences using SplitsTree4 v. 4.10 (Huson and Bryant 2006) (Fig. S2). Representative sequences of our 30 OTUs are archived at NCBI (Table S3). We calculated OTU dominance by dividing the OTU sequencing number with the total number of sequences per sample.

### Statistical analysis

All statistical analyses were completed in R (R version 2.15.1; R Core Team 2012).

Our sampling design included multiple sites in Montana and Washington. To determine whether we collected enough samples at each site to fully characterize the AMF community, we used species resampling curves (“specaccum” function, vegan package; Oksanen et al. 2011). We chose the “exact” method, which resamples with replacement, because sampling roots for AMF taxa are a technique equivalent to sampling sites (Gotelli and Colwell 2001). These curves just begin to asymptote, indicating that our sampling effort captured the most abundant taxa, but likely did not capture all of the rare taxa (Fig. S3).

The community data were reduced to two dimensions via nonmetric multidimensional scaling with the “metaMDS” function using a euclidean dissimilarity matrix in the same vegan package of R. Data reduction produced very low stress (0.16), and the nonmetric *r*^2^ between the ordination distance and observed dissimilarity was 0.97. Clustering of samples in the ordination was further supported by a hierarchical cluster analysis of the same data, which produced four similar groups (“pamk” function, Kaufman and Rousseeuw 1990; in the “fpc” package, Hennig 2013). The environmental factors, region, site, host plant, invasive cover, native cover, soil texture, and soil nutrients (OM, pH, conductivity, P, K, Ca, Mg, S, Na), were fit to the ordination with “envfit” function, and significance was assessed via 1000 permutations.

Community indices were completed with “diversity” function in the same vegan package. Network visualizations were completed using the “plotweb” function in the bipartite package (version 1.18, Dormann et al. 2008, 2009). Correlation matrices (“spearman”), *t* tests (“*t*.test”), ANOVAs (“aov”), and post hoc tests (“TukeyHSD”) were completed in the “stats” package. Total AM colonization fractions were arcsine transformed prior to analyses to meet assumptions of normality. All other data sets met ANOVA assumptions of homogenous variances (*P* > 0.10, “levene.test”) in the “lawstat” package) and normal distributions (“qqnorm” and “qqline”) in the “stats” package.

## Results

### Do AMF abundance and community composition differ between native and exotic forbs?

To address this question, we evaluated AMF colonization and OTU dominance and diversity in the invaded *C. stoebe* and *P. recta* and native communities at the three sites on MPG Ranch in Montana. All forbs were heavily colonized with >60% of the observed root intersections containing AMF structures (Table 1), although natives were significantly less colonized than *C. stoebe* and *P. recta* (*t*_43_ = 4.31, *P* < 0.001). Total, arbuscular, and vesicular colonization data were analyzed with 3 × 3 fixed-factor ANOVAs (three plant communities × three sites and five replicates). There was a significant interaction between site and host plant (*F*_4,36_ = 7.50, *P* < 0.001), in which colonization was higher in *C. stoebe* and *P. recta* than native forbs at the MPG2 and MPG3 sites, but there was no difference in colonization between host species at MPG1. The density of arbuscules (i.e., fraction of colonized intersections with arbuscules) varied only with host plant (*F*_2,36_ = 13.5, *P* < 0.001) and accounted for 50% of colonization in native forbs, which was 16% greater than in *C. stoebe* and 23% greater than in *P. recta* (Tukey's HSD, both *P* < 0.001, Table 1). The proportion of root intersections containing vesicles averaged 12% ± 7% (average ± standard deviation) and did not vary with site (*F*_2,36_ = 0.417, *P* = 0.662) or plant (*F*_2,36_ = 0.128, *P* = 0.881, Table 1).

We identified 25 OTUs across the native and invaded communities in Montana. All forbs were dominated by the same two taxa; OTU 1 and 16 (which belong to the *Glomus microaggregatum* and *Rhizophagus irregularis* clades, respectively).

### Do native plants have altered AMF associations in the presence of exotic plants?

Data on AMF colonization and OTU community metrics in the transition areas of the three Montana sites were used to address this question. All plants in the transition areas were highly colonized with an average AM colonization of 81% ± 14%. Total, arbuscular, and vesicular colonization data were analyzed with 2 × 3 fixed-factor ANOVAs (two plant communities × three sites and five replicates). There was a trend of higher colonization but a lower occurrence of arbuscules (Tukey's HSD, *P* = 0.063 and *P* = 0.021, respectively) in *P. recta* compared with native forbs. Colonization was higher overall in plants from the MPG2 site than MPG3 and MPG1 (Tukey's HSD, *P* = 0.010 and *P* = 0.045, respectively).

**Figure 1:**
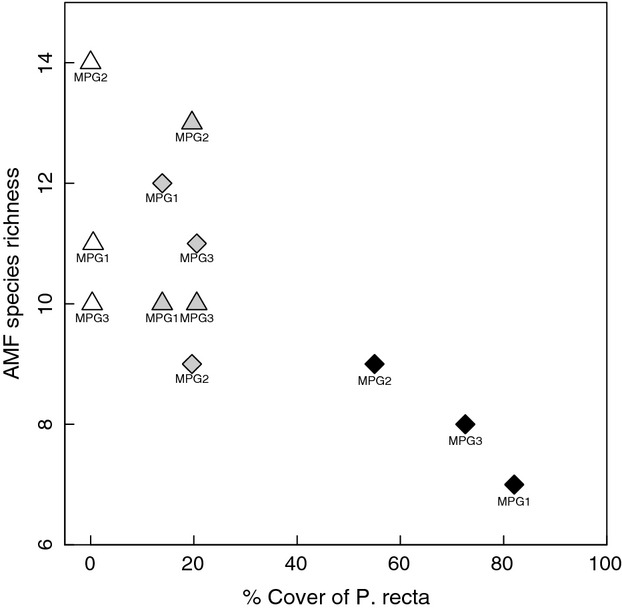
AMF species (OTU) richness as a function of percent cover of invading *Potentilla recta* at the three Montana sites (MPG1–3). Host plant is indicated by symbols; native forbs are represented by triangles, *P. recta* are represented by diamonds. Plant community is indicated by shading; the white symbols are native forb communities, the shaded symbols transition areas, and the black symbols are *P. recta* invasions.

**Figure 2:**
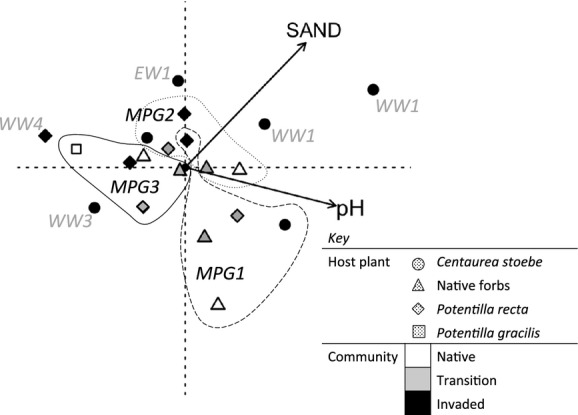
Ordination of AMF OTUs with significant environmental parameters (*P* ≤ 0.01, note that silt was inversely correlated with sand and is not shown here), OTU (not shown) and sample locations were plotted using weighted averaging. OTU assemblages of *Centaurea stoebe* roots (black circles) were divergent while the Montana *Potentilla recta* (diamonds) and native forbs (triangles) were more similar. Additionally, MPG1 and MPG2 transition areas (grey shading) had fungal communities that were intermediate between the native and invaded areas.

**Figure 3:**
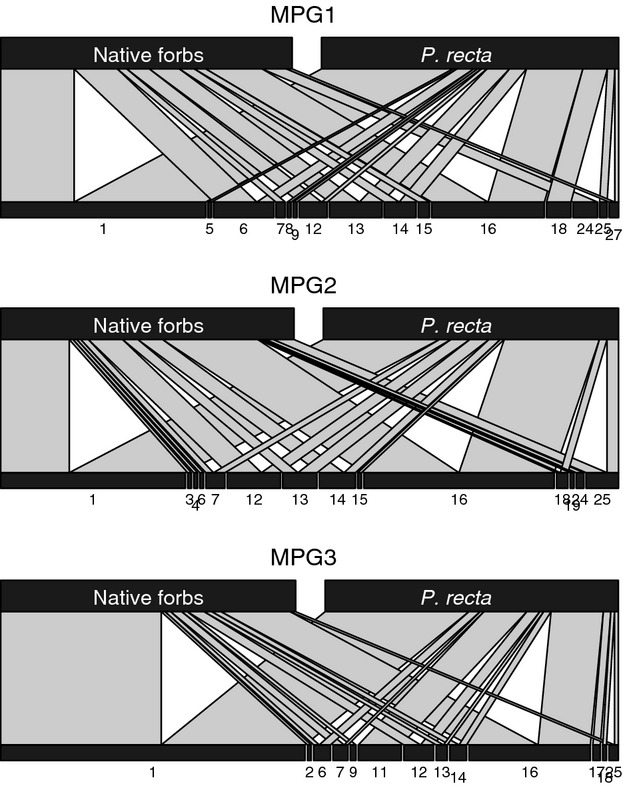
Bipartite network diagram of each transition plant community OTU distributions (bottom bars) in native forbs and *Potentilla recta* (top bars). The width of each connecting line indicates the relative abundance of the OTU in each host plant.

Dominance of OTU 1 and OTU 16 remained high in the transition areas, but fungal richness and diversity values in both *P. recta* and native forbs were intermediate between the high diversity observed in the native communities and the low diversity observed in the *P. recta*-invasions (Table 1). To understand the relationship between invasive cover (or invasion density) and the richness and diversity of the AMF communities, we examined the transition communities along with native and *P. recta* invaded communities. We found a negative correlation between *P. recta* cover and AMF richness (*r* = −0.70, *P* = 0.011) and diversity (*r* = −0.75, *P* < 0.001, Fig. 1). The loss of rare taxa with *P. recta* cover resulted in an AMF community that was a subset of the original, native AMF community and intermediate AMF communities in the transition zones between the invaded and native plots in two of the three sites (Fig. 2). Of the 21 OTUs present in these neighboring plants, 47%, 57%, and 62% of the taxa were present in both the *P. recta* and native forbs at MPG1, MPG2, and MPG3, respectively. Further, these common taxa accounted for over 80% of the AMF abundance in each host (Fig. 3).

### Do AMF abundance and/or community composition differ in areas where exotic plants are highly invasive and areas where they are not?

By examining the data collected from the five Washington sites along with data collected from the *P. recta* and *C. stoebe* invaded plant communities in the Montana sites, we were able to address this question. Total, arbuscular, and vesicular colonization data were analyzed with fractional 2 × 8 ANOVAs (two plant communities × eight sites and five replicates in 11 of 16 factor-level combinations, missing combinations were *C. stoebe* in WW4 and *P.recta* in WW1, WW2, WW3, and EW1). AM colonization varied by site (*F*_2,50_ = 7.88, *P* < 0.001); overall we found higher AM colonization in Montana and Eastern Washington than Western Washington (*t*_48_ = 3.70 *P* < 0.001, *t*_23_ = 2.36 *P* = 0.027, respectively, Table 1). There was no difference in AM colonization between *C. stoebe* and *P. recta* plants (*F*_1,44_ = 0.208, *P* = 0.651). Although our sample numbers are too low for a conclusive result (Fig. S3), it appears that the AMF community is typically dominated by OTU 1 and 16, even across the climate and invasion gradient. *P. recta* maintained fewer but a more consistent mix of rare AMF taxa than *C. stoebe*. The singular Washington *P. recta* sample from WW4 stands out as different (Fig. 2) due to the low richness (6; Table 1) and lack of OTU 1. The WW4 sample clustered with the native *P. gracilis* sample (Table S4). In contrast, *C. stoebe* had a wide and consistent variation in rare taxa across sites (Fig. 2).

### What are the main drivers of AMF communities at these sites?

We found a total of 30 OTUs across all plant communities (Fig. S2), with individual plant communities harboring anywhere from 6 to 14 OTUs. AMF communities were best predicted by soil texture (fine sand and silt) and pH (all *P* < 0.01; Fig. 2), not host plant community. Other significant predictors, including soil nutrients and site (*P* < 0.05), were covariates of texture and pH. The AMF communities from the sites in Montana were similar to each other, clustering near the center of the ordination, while the AMF communities from the sites in eastern and western Washington were divergent (Fig. 2). This result must be considered in light of the fact that four of the five Washington samples were taken from *C. stoebe* invasions.

While the categorical plant communities (*C. stoebe* invasion, *P. recta* invasion, native forb, transition zones) were not a significant predictor of OTU assemblage, invasion density (i.e.,% cover by exotics) correlated positively with dominance by two most abundant OTUs (OTU 1 and OTU 16) and negatively with OTU richness. Dominance of OTU 1 and 16 increased from 12% to 78% with increasing cover of invasive species (spearman's rank correlation: *r* = 0.57, *P* = 0.010), while AMF richness decreased from 14 to 6 OTUs with increasing cover of invasive species (spearman's rank correlation: *r* = −0.61, *P* = 0.006). *P. recta* and *C. stoebe* differed in the strength of these relationships. *P. recta* were dominated by OTU 1 and OTU 16 across the invasion gradient, but lost species richness at higher densities, the exception to this trend was *P. recta* from WW4 in western Washington which had low richness even at low invasion density. *C. stoebe* maintained species richness, but had a large increase in OTU 1 and OTU 16 dominance with increasing cover. Additionally, the rare taxa in each *C. stoebe* sample varied significantly; of the 30 OTUs detected in *C. stoebe* roots, each site only hosted 8–11 different AMF taxa.

## Discussion

Using survey data, we assessed the potential of AMF to influence invasion success. We specifically asked (1) whether native and exotic forbs at the same sites harbor different AM colonization and AMF communities, (2) whether the composition of AMF communities in native forbs is different in the presence of exotic forbs, and (3) whether exotic forbs harbor different AM colonization and AMF communities across regions (and simultaneously across a gradient of invasion density). The nature of the data sampled also allowed us to assess whether the AMF community composition correlated more strongly with the soil environment, plant communities, or spatial distance among sites.

Previous comparisons between native and exotic invasive plants have focused on specific native species with selected invaders and found significant shifts in AMF communities associated with native plants when co-occurring with invaders (Mummey et al. 2005; Hawkes et al. 2006; Stinson et al. 2006; Busby et al. 2011). However, generalizing those results to apply broadly to invasive species may be problematic because comparisons included different plant functional groups, which is arguably the most important factor in predicting mycorrhizal effects (Hoeksema et al. 2010; Lekberg et al. 2013). To minimize confounding factors, we focused our study on non-N_2_-fixing, mycorrhizal forbs across multiple, independent invasions to isolate the effect of plant provenance from factors of mycorrhizal status and plant functional group.

### Do AMF abundance and community composition differ between native and exotic forbs?

All forbs, exotic and native, were highly colonized with rates ranging from 61% to 96%. High rates of colonization may imply a high degree of mycorrhizal dependency (Hetrick et al. 1996; Wilson and Hartnett 1998), even in the invasive plants *C. stoebe* and *P. recta*, which conflicts with previous suggestions that many invasive plants have a low or evolved mycorrhizal dependency (Seifert et al. 2009; Pringle et al. 2009; Vogelsang and Bever 2009; but see Klironomos 2002 and Shah et al. 2008). Indeed, several studies have shown that *C. stoebe* depend on AMF to be competitive (Marler et al. 1999; Callaway et al. 2004).

The greater relative abundance of arbuscules in native compared with exotic forbs (Table 1) could indicate increased nutrient exchange between plant and fungus (Smith and Read 2010). More arbuscules in native forbs may also reflect some degree of preferential resource allocation between plants and fungi, because arbuscules (and the analogous hyphal coils) are the primary symbiotic interface across which nutrients are transferred (Smith et al. 2001; Smith and Smith 2011), and more arbuscules have been observed in hosts that deliver more carbon (Lekberg et al. 2010). Hausmann and Hawkes (2009) observed a similar increase in arbuscules in a greenhouse experiment of native and exotic grasses and concluded that AMF prefer native hosts. However, our study is based on a one-time sampling and differences may also be attributed to seasonal growth patterns of plants. A more focused study on costs and benefits across seasons is required to determine whether arbuscule abundance is greater in native plants year round, and to assess possible consequences for mycorrhizal function.

Although richness of AMF taxa in roots is greater than once believed (Öpik et al. 2006), plant roots are typically dominated by just a few taxa that can account for 40% of all recovered sequences (Dumbrell et al. 2010). We observed the same pattern here; all but two of the seventeen plant communities sampled (both native and invasive) were dominated by two OTUs (OTU 1 and 16, closely related to *Glomus microaggregatum* and *Rhizaphagus irregularis,* respectively), which accounted for 54% of all sequences recovered in this study. By considering the invasion gradient, we can infer how invading *P. recta* interacts with existing AM. In the pre-invasion (native community), OTU 1 and 16 made up 44% of the recovered sequences, but this increased to 62% of recovered sequences in the “post-invasion” (exotic monoculture community). These data suggest that this exotic forb forms symbioses with pre-existing dominant AMF rather than promoting rare AMF taxa. Whether or not these OTUs contribute to invasiveness or respond to an increased abundance of a preferred host is unknown and will require manipulating OTU abundances. It is interesting to note, however, that both *Rhizophagus irregularis* and *Glomus microaggregatum* are considered generalists in their host and environmental preferences, which is consistent with suggestions that exotic plants may associate with generalist, cosmopolitan mutualists to a greater extent than native plants (Dickie et al. 2010; Moora et al. 2011; Öpik and Moora 2012).

The greater dominance of OTU 1 and 16 in *P. recta* relative to native forbs was coupled, not unexpectedly, with a reduced AMF richness. Lower richness might be expected because we sampled only a single plant species within invasions but included multiple species in native plant communities. At least some host preference exists in the AM symbiosis (e.g., Vandenkoornhuyse et al. 2003), and plant species richness correlates with AMF species richness under at least some conditions (van der Heijden et al. 1998; Landis et al. 2004). However, we did not see a reduced AMF richness in the *C. stoebe* invasions, which corroborates earlier findings (Lekberg et al. 2013) and shows that richness can be high in monodominant stands of mycotrophic invasive forbs. *P. recta* and *C. stoebe* affected AMF richness differently, yet they had similar biomass production (Gallagher 2011) and AM colonization – two factors that may correlate with belowground C-allocation and in turn overall fungal richness (Waldrop et al. 2006). These apparently inconsistent results indicate that more work is required to determine the mechanisms by which host plants affect AMF richness.

### Do native plants have altered AMF associations in the presence of exotic plants?

Altering the AMF of native neighbors may be one mechanism by which exotic forbs increase competitiveness (Zhang et al. 2010). *C. stoebe* can alter the mycorrhizae of adjacent *Pseudoroegneria spicata* (bluebunch wheatgrass plants; Mummey et al. 2005), and exotic annual grasses can decrease AMF richness in co-occurring native perennial grasses (Hawkes et al. 2006). In sampling across the three *P. recta* invasion gradients, we observed only a small shift in AMF communities. Some rare taxa were lost during invasion, but OTU 1 and 16 remained dominant (Table 1). In two of our three sites (MPG1 and MPG2), the AMF communities found in the roots of *P. recta* and the native forbs growing in the transition area were both intermediate to those found in the “pre-invasion” (native) community and the “postinvasion” monoculture. Apparently, neighboring plants of the same functional group can influence each other's AMF communities. However, whether these shifts are significant enough to affect overall mycorrhizal function is debatable and will require more detailed *in situ* functional studies.

Neighboring *P. recta* and native forbs shared both dominant and rare OTUs (Fig. 3). The redundancy in AMF taxa and the close proximity of these plants (<10 cm) make it highly likely that these plants were connected via common mycorrhizal networks (CMNs). Nutrients such as nitrogen (He et al. 2009) and phosphorus (Wilson et al. 2006) can be transferred through CMNs, and the terms of carbon/nutrient trade between fungi and host plant have important implications for plant competitive interactions. Careful *in vitro* studies have found convincing evidence for bidirectional control of the carbon/nutrient trade between plants and fungi (Lekberg et al. 2010; Hammer et al. 2011; Kiers et al. 2011; Fellbaum et al. 2012). If exchange is truly “tit for tat,” then CMNs may create a positive feedback where larger hosts contribute more carbon and receive more benefit than their smaller neighbors (Weremijewicz and Janos 2013; but see Walder et al. 2012). This alone could help explain the success of some fast growing exotic species. Further, some plant-AMF species combinations are more beneficial to host plants than others (Klironomos 2002, Kiers et al. 2011; Walder et al. 2012), and exotic competitiveness could increase if exotic plants are capable of shifting AMF communities toward more cooperative AMF taxa, although we did not find evidence of this. Finally, there may be temporal differences in exchange, which have not yet been explored, but are critical to understanding the implications of CMNs for invasion success across relevant time periods.

### Do AMF abundance and/or community composition differ in areas where exotic plants are highly invasive and areas where they are not?

Arbuscular mycorrhizal fungi abundance and community composition of two exotic forbs were compared between regions where these exotics are highly and moderately invasive, similar to the biogeographical approach taken by previous investigators to identify drivers of invasions (e.g., Callaway et al. 2004; Lankau 2010). We hypothesized that if AMF influence plant invasions, then AMF abundance and/or the community composition would differ between areas where the forbs are highly invasive (Montana) and moderately invasive areas (western Washington). We found that AM colonization tended to be higher in Montana plants, but all exotic forbs were highly colonized (>60%), and we found no strong shifts in AMF community composition. Thus, while some of these changes were statistically significant, it is unclear whether they are functionally important.

### Overall drivers of AMF community assembly

The ordination of our data from all sites suggests that soil abiotic factors were more important than host plant identity and site for determining AMF community composition when plants from the same functional group were compared. This agrees with previous work and reinforces the importance of pH and texture (Fierer and Jackson 2006; Lekberg et al. 2007) as important environmental habitat filters for microbial community assembly. Of the 30 OTUs detected, each plant community only hosted between 6 and 14 different OTUs and most were dominated by two OTUs that appear to have global distributions. Genetic dominance does not necessarily translate into relative abundance in roots however. Structure formation differs between AMF genera (Dodd et al. 2000); and a higher density of vesicles, which contain thousands of copies of nuclei (Bécard and Pfeffer 1993), can result in a higher number of sequences per unit fungal biomass. Both *G. micoraggregatum* and *R. irregularis* typically produce large numbers of vesicles, which may at least partially explain their dominance throughout this data set. Overall vesicle formation was consistent across all plant communities, however, so shifts in dominance from *P. recta* to native forbs are likely to reflect shifts in relative abundance of taxa in roots. The overall richness found here is lower than was found in a similar study by Lekberg et al. (2013), but we used substantially fewer sequences, and thus, our focus here was on dominant OTUs rather than an exhaustive characterization of all taxa present. It is also interesting to note that site-specific taxa tended to be rare, which agrees with Öpik et al. (2006).

### Summary

Utilizing survey data that can reveal the end result of long-term ecological processes, we observed greater mycorrhizal colonization in exotic than in native forbs, and in regions where exotics are highly invasive. This could imply that AMF directly benefit exotic plants and may contribute to invasions, although natives were only slightly less colonized and fungal abundance alone seems an unlikely explanation for invasive success. Samples collected along invasion gradients suggested that *P. recta* joins existing mycorrhizal networks, simultaneously increasing dominance of the two most abundant OTUs and decreasing richness of rare taxa. If rare and specialist taxa are important for native forbs, this decrease in richness may indirectly benefit *P. recta* by decreasing native forb competitiveness. But again, the shifts were small and may not be biologically significant. Also, there were only slight differences in AMF abundance and community composition in areas where *P. recta* and *C. stoebe* are in high abundance compared with areas where they are not, which appears inconsistent with a strong, mycorrhizal-mediated invasion. Across our study, community assemblages were best predicted by abiotic parameters (soil pH and texture), not plant community type, adding to the growing body of evidence that some taxa are widespread generalists and environmental conditions are important filters. While controlled experiments are required to verify the functional consequences of the patterns observed here, we found only weak support for mycorrhizal-mediated invasions of *C. stoebe* and *P. recta* in the intermountain west.

## Data Accessibility

Native forb species list Table S1. Phylogenetic data: National Center for Biotechnology Information (NCBI) Read Archive accession numbers in Table S3. Other data will be archived upon acceptance.

## Supporting Information

**Figure S1.** OTU accumulation curve **Figure S2.** Neighbor net of fungal OTUs **Figure S3.** Sample effort curve

**Table S1.** Native plant species sampled in Montana **Table S2.** Soil abiotic factors **Table S3.** OTU accession numbers **Table S4.** OTU relative read numbers.
