# Supplementary material for: Grassland invaders and their mycorrhizal symbionts: a study across climate and invasion gradients
Source: Ecol Evol. 2014 Feb 19;4(6):794–805. doi: 10.1002/ece3.917 (PMC3967904; doi:10.1002/ece3.917)

Figure S1. OTU accumulation curves for each site indicate that our extraction and amplification protocol capture the majority of OTUs present even in our smallest sample of 112 AMF “individuals.”


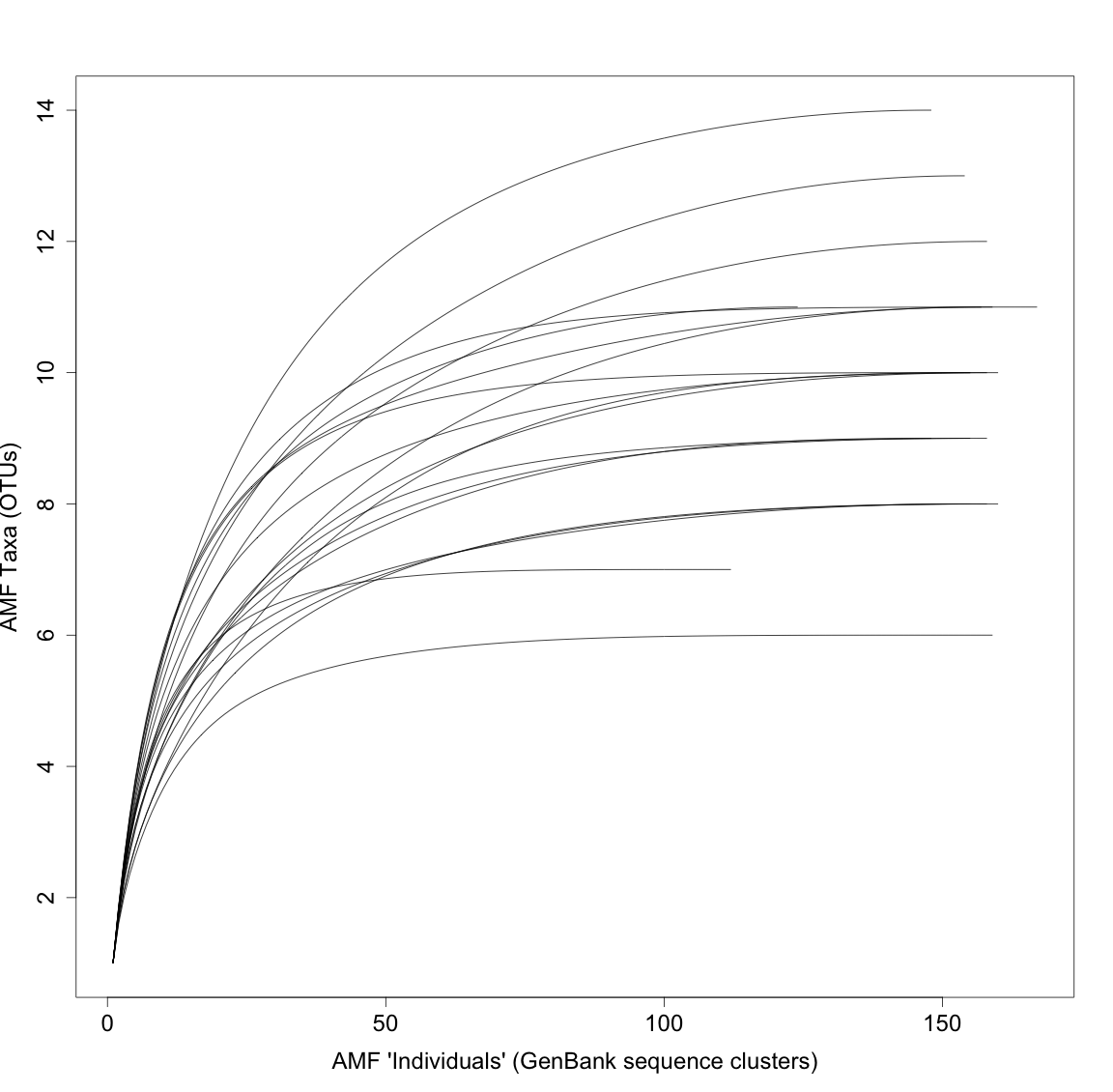


Figure S2. NeighborNet split network based on least square distances of LSU rDNA sequences within the Glomeromycota. The circles indicate operational taxonomic units (OTUs) defined as monophyletic clades. OTU 1-7 represents groups related to *Glomus microaggregatum* (green circle), OTU 12, 13, 15, 16 and 17 represents OTUs that encompass *Rhizophagus irregularis* (red circle) and *Funneliformis mosseae* is included in OTU 24. *Claroidoglomus* is found in OTU 25-29.


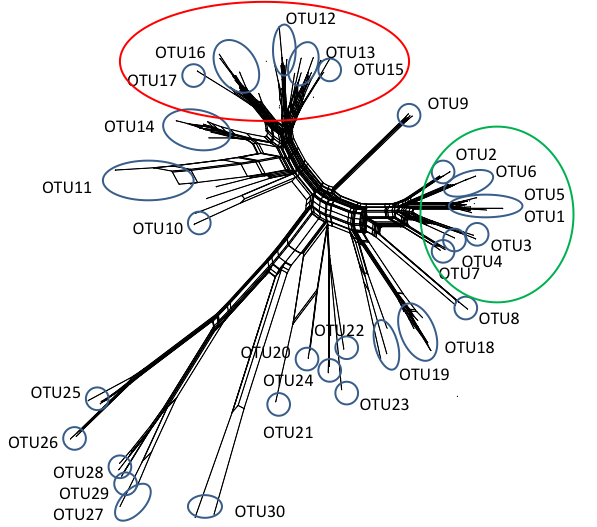


Figure S3. Sampling effort curves for the three subsets of the data: Washington and Montana exotics, *P. recta* and *C. stoebe* (dark grey 95% confidence interval), the Montana natives and exotics, *P. recta* and *C. stoebe* (white 95% confidence interval), and the Montana Transition areas with neighboring natives and *P.recta* (light gray 95% confidence interval).


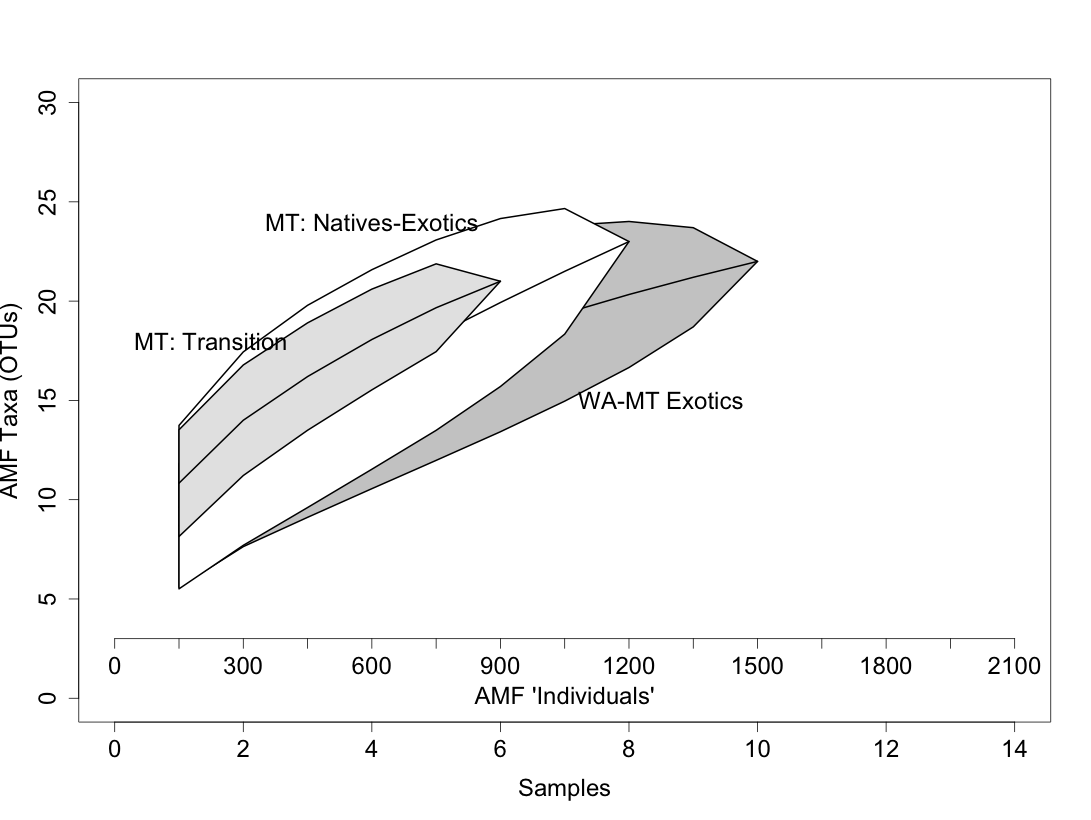

Supplement: Supplementary file 1 — Figure S1. OTU accumulation curve Figure S2. Neighbor net of fungal OTUs Figure S3. Sample effort curve [file ece30004-0794-sd1.docx]
